# Postoperative Autologous Reinfusion in Total Knee Replacement

**DOI:** 10.1155/2015/826790

**Published:** 2015-09-09

**Authors:** A. Crescibene, F. Martire, P. Gigliotti, A. Rende, M. Candela

**Affiliations:** ^1^Orthopedics and Traumatology Unit, San Francesco di Paola Hospital, Azienda Sanitaria Provinciale di Cosenza, Via Promintesta, 87027 Paola, Italy; ^2^Kidney and Transplantation Research Center, Azienda Ospedaliera di Cosenza, Via Felice Migliori, 87100 Cosenza, Italy; ^3^Transfusion Medicine Unit, San Francesco di Paola Hospital, Azienda Sanitaria Provinciale di Cosenza, Via Promintesta, 87027 Paola, Italy

## Abstract

Surgeries for total knee replacement (TKR) are increasing and in this context there is a need to develop new protocols for management and use of blood transfusion therapy. Autologous blood reduces the need for allogeneic blood transfusion and the aim of the present study was to verify the safety and the clinical efficacy. An observational retrospective study has been conducted on 124 patients, undergoing cemented total knee prosthesis replacement. Observed population was stratified into two groups: the first group received reinfusion of autologous blood collected in the postoperative surgery and the second group did not receive autologous blood reinfusion. Analysis of data shows that patients undergoing autologous blood reinfusion received less homologous blood bags (10.6% versus 30%; *p* = 0.08) and reduced days of hospitalization (7.88 ± 0.7 days versus 8.96 ± 2.47 days for the control group; *p* = 0.03). Microbiological tests were negative in all postoperatively salvaged and reinfused units. Our results emphasize the effectiveness of this procedure and have the characteristics of simplicity, low cost (€97.53 versus €103.79; *p* < 0.01), and easy reproducibility. Use of autologous drainage system postoperatively is a procedure that allows reducing transfusion of homologous blood bags in patients undergoing TKR.

## 1. Introduction

Total joint replacement usually represents the final route for treatment of degenerative disease of the knee. Joint replacements are more and more frequent and routine these days thanks to, for example, the consequent aging of the general population, increased functional requirements, and the development and use of new materials and more sophisticated surgical techniques.

In this context, there is a need to develop new protocols for management and use of transfusion therapies in the field of orthopedic surgery.

Transfusion of homologous blood are frequent and costly and expose patients to potential risks of infection [1, 2]; hence several methods have been proposed to avoid them [3, 4].

Preoperative blood donation and intra- and postoperative blood collection and administration of pharmaceutical agents to either reduce blood loss (e.g., tranexamic acid) or stimulate the production of erythrocytes (e.g., erythropoietin) have been proposed as alternative techniques to transfusion of homologous blood in various studies [3, 4].

In the scientific literature, there are several reports of studies where reinfusion of blood collection in postoperative orthopedic surgery, especially in prosthesis surgery [5–14], is analyzed.

The aim of the present study was to verify the safety, the clinical efficacy, and the possible benefits of reinfusion of postoperatively collected autologous blood in total knee replacement procedures, with special emphasis on cost-benefit and reinfusion procedure, by comparing autologous blood transfusion with nonautologous blood transfusion.

## 2. Materials and Methods

### 2.1. Patients

Between 2011 and 2012, one hundred twenty-four patients with a mean age of 71.2 ± 6.8 years and a range between 50 and 84 years were included in the study.

All patients were diagnosed with primary osteoarthritis of the knee.

The first group consisted of a series of sixty-four consecutive patients (male to female ratio 1 : 5, range of age between 62 and 84 years), who underwent one stage unilateral total knee arthroplasty using a blood reinfusion device in 2012.

Fifty-eight of these patients received autologous transfusion and six patients were excluded, five due to systemic pathologies.

The control group consisted of sixty consecutive patients (male to female ratio 1 : 3, age ranging from 50 to 79 years). This population was operated on consecutively in 2011 but is not subjected to autologous blood reinfusion because we have been using this device since January 1, 2012.

Before any study-related measures were taken all patients read and signed informed consent for any possible transfusion of homologous red blood cell and a specific consent for reinfusion, after adequate information on possible risks and benefits of both methods.

The manuscript was performed in accordance with the ethical standards of the 1964 Declaration of Helsinki as revised in 2000; for this type of study formal consent is not required.

### 2.2. Surgical Procedure

Antibiotic prophylaxis was obtained by administration of ceftriaxone 1 g intramuscular injection once daily and teicoplanin 800 mg intravenous injection once daily, 30 minutes before inducing anaesthesia.

Antithrombotic prophylaxis was obtained by administration of enoxaparin sodium 4,000 I.U. once daily by subcutaneous injection for 35 days, 12 hours before surgery; two patients had an urticarial reaction and therefore it has been replaced with fondaparinux sodium 2.5 mg once daily by subcutaneous injection for 35 days.

Patients were subjected to surgical intervention for unilateral primary arthroplasty knee, by implanting a Zimmer's Nexgen cemented prosthesis or How Medical's Triathlon prosthesis.

All patients received subarachnoid anesthesia.

Surgery was carried out by the same surgeon on all patients; a standard surgical procedure was performed including a longitudinal medial skin incision and median parapatellar quadriceps splitting approach.

The hole drilled in the femoral canal was plugged.

Limb ischemia was achieved through temporary leg loop tire located at the root of the limb and the tourniquet was deflated after the step of cementing.

Postoperative pain was controlled with paracetamol 100 mL/10 mg endovenous with a 4-hour minimum interval between each administration.

### 2.3. Blood Salvage and Reinfusion

Bellovac ABT is a drainage system for postoperative collection, filtration, and reinfusion of shed autologous blood. It consists of all components required for collection and reinfusion of one unit of shed autologous blood.

The initial negative suction pressure is 90 mmHg (12 kPa), generating safe and efficient drainage.

Once the recovery of shed blood is completed (within <6 hours) the transfusion bag can be replaced with a collection bag for evacuation only, thus serving as a simple wound drainage (Card System Technical Bellovac ABT for drainage and the recovery of the blood postoperative manufacturer: Astra Tech AB, Via Cristoni, Casalecchio di Reno (BO)).

The minimal drained blood to be transfused is 100 cc.

### 2.4. Transfusion Trigger

For all patients, we decided that a homologous transfusion therapy was only indicated for haemoglobin values below 8 g/dL [15].

### 2.5. Clinical Data

The study group consisted of a series of sixty-four consecutive patients with mean age 73.3 ± 5.7 years (14 males and 50 females), while the control group consisted of sixty consecutive patients with mean age 69 ± 7.2 years (18 males and 42 females).

We excluded patients with systemic pathologies such as uncompensated diabetes mellitus, cancer, severe cardiovascular pathologies, immunodepression, anticoagulating or antiaggregating therapy, coagulation disorders including deep venous thrombosis, and ongoing infections.

We considered as discriminating factors hemoglobin values <12 g/dL for women and <14 g/dL for men.

Length of surgery was 2.24 ± 0.38 hours in the study group versus 2.06 ± 0.32 hours (*p* = 0.17).

### 2.6. Laboratory Data

Hemoglobin values (Hb; g/dL) were checked preoperatively, immediately after surgery, on the first day and the second day after the operation, and on discharge. They were later compared between groups.

In the study group, patients were subjected to a microbiological culture of a blood sample (20 mL) from the bag of postoperative recovery, at the end of the procedure of reinfusion.

### 2.7. Economic Data

In our hospital, the transfusion medicine unite has provided the following expense items: the cost of an autologous blood retransfusion system is around €68,00, while the costs of an allogenic blood transfusion, including cross-matching, delivery, and refrigerated storage, are stated to be €270.

Added costs of the postoperative drain and the ABT system are €20 and €68, respectively.

These costs were evaluated for the examined patients.

### 2.8. Statistic

All data are presented as mean ± SD or median (IQR) as appropriate. Groups were compared using the one-way ANOVA or *t*-test for normally distributed data and the nonparametric Kruskal-Wallis or Mann-Whitney *U* test for non-normally distributed variables.

## 3. Results

### 3.1. Preoperative Comparison of Groups Characteristics

The study group consisted of a series of sixty-four consecutive patients with mean age 73.3 ± 5.7 years, 14 male and 50 female, while the control group consisted of sixty consecutive patients with mean age 69 ± 7.2 years, 18 male and 42 female (*p* < 0.05).

The average body weight of the study group was 70.8 ± 8.8 Kg and 73.1 ± 8.0 Kg (*p* = 0.43).

### 3.2. Perioperative Data

In the study group, all the patients received autologous blood transfusion.

In the first six hours 400 ± 122.4 mL (min. 100; max. 600) was collected and retransfused; within 24 hours there was 198.5 ± 142.4 mL of blood drained in the drainage.

In the control group, 478.2 ± 220.3 mL (min. 250; max. 900) was drained in the first 24 hours after surgery.

Because of the intraoperative use of a tourniquet, intraoperative blood loss was negligible in both groups.

We noticed a reduction rate of allogeneic blood transfusion between the study and the control group (10.3% versus 30%; *p* = 0.08).

Six patients in the study group, four women and two men, were subjected to allogeneic transfusion, five patients received one unit of homologous blood between the second and fourth days postoperatively, and one patient received two units of homologous blood. No patient had any adverse event or febrile episodes.

In the control group, we registered 18 patients who received homologous blood transfusion; each one had received a bag of homologous whole blood.

The transfusion trigger in the ABT group (*n* = 6) was 7.5 ± 0.1 g/dL, whereas that in the control group (*n* = 18) was 7.3 ± 0.2 g/dL (*p* = 0.09).

One unit of red blood cells can be expected to result in a hemoglobin increase of 1 g/dL in a typical adult [15].

In the ABT group we recorded an increase in hemoglobin equal to 0.7 ± 0.3 (g/dL), after autologous transfusion. In this group we recorded an increase in hemoglobin equal to 1.3 ± 0.2 (g/dL) (*n* = 7 bags) versus 0.9 ± 0.1 (g/dL) (*n* = 18 bags) in the control group after homologous transfusion (*p* < 0.01).

**Figure 1:**
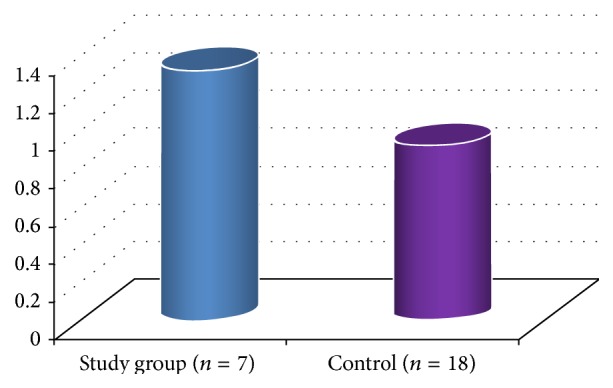
Hemoglobin increase after homologous transfusion (g/dL).

Therefore, we noticed a greater rise in hemoglobin after homologous transfusion in the group given reinfusion (Figure 1).

The tourniquet time was 1.13 ± 0.27 hours in study group versus 1.04 ± 0.27 hours in control group (*p* = 0.36).

In the study group the mean length of stay at the hospital (LOS) was 7.88 ± 0.7 days, while it was 8.96 ± 2.47 days for the control group (*p* = 0.03).

Blood values were substantially stationary in the perioperative phase, in either group.

**Figure 2:**
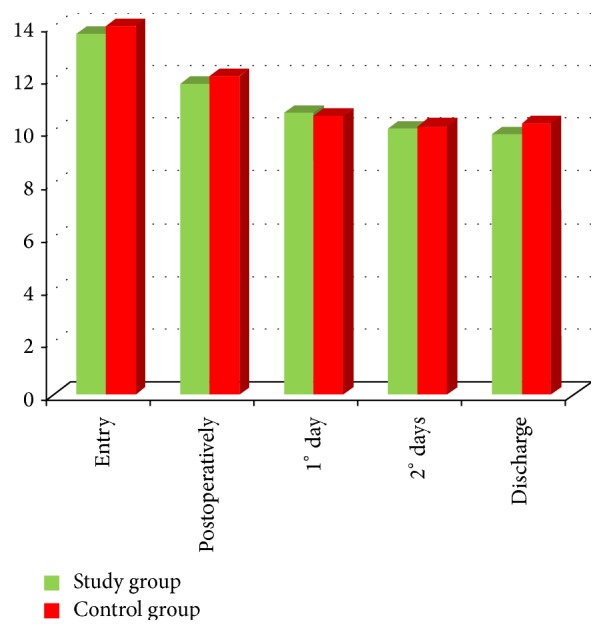
Modification of hemoglobin level in both groups during stay at the hospital period (g/dL).

**Figure 3:**
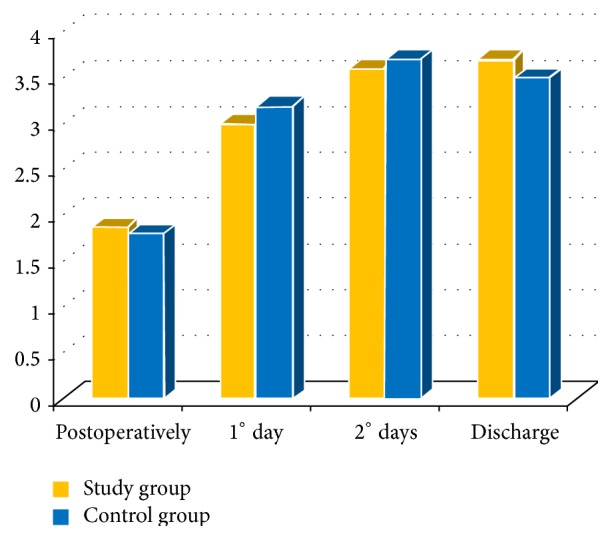
Changes in hemoglobin level in both groups during stay at the hospital period (g/dL).

Really, we did not find any significant difference for hemoglobin count between groups from admission to hospital discharge (Table 1 and Figure 2); as expected, we did not find any difference between groups considering the reduction in hemoglobin for study's time points (Table 2 and Figure 3).

### 3.3. Complication and LOS

The results of microbiological cultures performed on blood samples taken from the postoperative blood bag at the end of the procedure of reinfusion were negative in all cases.

In our study a total knee replacement surgical procedure, along with the complementary use of a device for postoperative collection and reinfusion of shed blood resulted in an average reduction of the length of hospital stay equal to 1.08 ± 0.76 days (*p* = 0.03 versus control).

### 3.4. Cost Analysis

The cost of an autologous blood retransfusion system is around €68,00, while the costs of a allogeneic blood transfusion are stated to be €270, including cross-matching, delivery, and refrigerated storage.

In the present study, 18 allogeneic transfusions were given in the no-drainage group, while 7 allogeneic transfusions were given in the ABT group, at a cost of €4.860,00 and €1.890,00, respectively.

The additional costs of the postoperative drain and the ABT system were €20 and €68, respectively, in either group at a cost of €1.160,00 (*n* = 58) and €4.352,00 (*n* = 64).

The average cost per patient turned out to be lower in the group receiving reinfusion (€97.53 versus €103.79; *p* < 0.01).

## 4. Discussion

Primarily, autologous blood reduces the need for allogeneic blood transfusion; furthermore, it prevents the transmission of viral diseases (hepatitis C virus, hepatitis B virus, human immunodeficiency virus, and Creutzfeldt-Jacob virus), transfusion reactions, and transfusion errors [16, 17].

The main advantage of postoperative collection and reinfusion of shed blood is this method's simplicity; hence it finds its application in traumatized patients.

However, its main disadvantage is the risk of contamination during blood collection [8, 18].

Fong et al. [19] identify some possible complications of collection of red blood cells, such as nonimmunologic haemolysis, gas embolism, nonhaemolytic transfusion reaction, coagulopathies, contamination with drugs, the intraoperative use of washing solutions in the surgical site and infectious agents, cytokines, and other microaggregates.

The risk of complications decreased due to improvement of techniques and practices as well as increased experience with autologous blood collection systems [20].

Cleveland Clinic has performed a five-year retrospective study of complications, with both homologous and collected shed blood, noting that the incidence of complications with collected blood was around 0.027% compared to 0.14% of homologous blood [21].

In our study we had no patient with adverse events or febrile illnesses.

The results of microbiological cultures performed on blood samples taken from the postoperative blood bag at the end of the reinfusion were negative in all cases.

The purpose of collection of shed blood is to reduce or eliminate the need for homologous blood transfusion and the associated risk of infectious and noninfectious complications [22].

During 2006, a meta-analysis of the recovery of red blood cells in adult elective surgery showed that recovery of blood could reduce the need for homologous blood transfusion.

The use of recovered red blood cells has reduced the exposure to allogeneic blood by 39% with an average saving of 0.67 units per patient [23].

In 2013 a meta-analysis proved that the use of a postoperative autotransfusion reinfusion system reduced significantly the demand for allogeneic blood transfusions.

It also cut the number of patients who needed allogeneic blood transfusions and the cost of hospitalization after total knee arthroplasty.

Collection of blood in orthopedic surgery results in greater safety and efficacy [24–26].

94 cases have been studied but a power analysis of the study was not performed; however, we argue that the use of a device for autologous blood collection and washing results in an average reduction of 4 units of allogeneic blood and cost savings of an average of 406.84 dollars per patient [27].

In the literature there are some authors with differing views.

These authors suggest that autologous blood transfusion drains have no effect on the proportion of transfused patients in primary total knee arthroplasty [28–34].

The postoperative hemoglobin levels, the length of hospital stay, and the adverse events are also comparable between groups [28].

In our opinion autologous blood collection and reinfusion, by the use of postoperative systems, is a procedure that allows limiting the transfusion of homologous blood to patients undergoing TKR, improving the postoperative course from a psychophysical point of view and allowing an early transfer to rehabilitation unit.

We noticed a reduction in allogenic blood transfusion between the study and the control group (10.3% versus 30%; *p* = 0.08).

Reinfusion group patients also displayed a reduction in the length of stay in hospital by 1.08 ± 0.76 days (*p* = 0.03 versus control).

Concerning costs, the question is whether savings derived from the reduction in allogeneic blood transfusion requirements outweighed the extra costs of the ABT system.

Zacharopoulos et al. [35] stated that use of the postoperative blood reinfusion systems is highly effective in reducing the demand for homologous blood transfusion for patients undergoing total knee replacement surgery, resulting in important cost savings in the management of these patients.

A previous study observing patients undergoing TKA found net savings in different cost scenarios of €5 to €106 per patient with the same ABT system as used in this study and €52 to €50 per patient with another ABT system [36].

A review of cost-effectiveness on blood-saving measures stated that cell salvage had lower costs compared with all of the alternative blood-saving strategies except acute normovolemic dilution and concluded that autotransfusion may be a cost-effective method to reduce allogeneic transfusions [37, 38].

The evidence that supports the use of red blood cell recovery in knee prosthesis is stronger and comprises randomized studies and one large retrospective review [39, 40].

The average cost per patient was found to be lower in the group receiving reinfusion (€97.53 versus €103.79; *p* < 0.01).

This work presents a lot of strengths such as the same characteristics of the sample, the surgical technique performed by the same surgeon, and the rigorous cost analysis; on the contrary we are aware of some weaknesses such as the retrospective design. So some prospective randomized studies are to be presented.

## 5. Conclusion

This study has confirmed the absolute safety of the device and the absence of bacteria in examined samples.

Our results emphasize the effectiveness of this procedure and have the characteristics of simplicity, low cost, easy reproducibility, and safety.

In addition, this recovery system replaces the simple postoperative surgical drainage, representing a further saving of economic resources.

Finally we conclude that the use of the shed blood recovery system Bellovac ABT constitutes a valid device, which can find wide application in orthopedics, especially in the context of total knee replacement surgery.

## Figures and Tables

**Table 1:** Values are presented as mean ± standard deviation (Hb; g/dL).

	Study group	Control group	*p* value
Entry	13.7 ± 1.5	14 ± 1.2	*p* = 0.12
Postoperatively	11.8 ± 1.22	12.1 ± 1.3	*p* = 0.11
1° day	10.7 ± 1.2	10.6 ± 1.2	*p* = 0.16
2° days	10.1 ± 1.1	10.2 ± 0.9	*p* = 0.28
Discharge	9.9 ± 1.1	10.3 ± 0.9	*p* = 0.14

**Table 2:** Reduction of blood parameters; values are presented as mean ± standard deviation.

	Study group (*n*. 52)	Control group (*n*. 42)	*p* value
Postoperatively	1.9 ± 0.8	1.8 ± 0.5	*p* = 0.22
1° day	3 ± 1	3.2 ± 0.9	*p* = 0.34
2° days	3.6 ± 1.4	3.7 ± 1	*p* = 0.21
Discharge	3.7 ± 1.1	3.5 ± 1.1	*p* = 0.21
